# Depression stigma and migration – results of a survey from Germany

**DOI:** 10.1186/s12888-017-1549-y

**Published:** 2017-11-29

**Authors:** Anna C. Makowski, Olaf von dem Knesebeck

**Affiliations:** Department of Medical Sociology, University Medical Center Hamburg-Eppendorf, Martinistrasse 52, 20146 Hamburg, Germany

**Keywords:** Public stigma, Self-stigma, Attitudes, Depression, Germany, Differences, Migrants, Non-migrants

## Abstract

**Background:**

There are barely any studies focusing on migration in relation to mental illness stigma. We explore present attitudes regarding depression among migrants (either born in Germany or born abroad) and non-migrants in Germany, drawing upon three components of public stigma: stereotypes, emotional reactions and desire for social distance. Furthermore, differences in self-stigma of depression between the two groups are analyzed.

**Methods:**

Analyses are based on a representative telephone survey (*N* = 2013) in Germany. Respondents were presented with a vignette depicting either someone from Turkey or from Germany affected by depression, followed by questions on stereotypes, emotional reactions and desire for social distance. The (anticipated) self-stigma of depression was also assessed. Analyses of variance tested for differences between migrant and non-migrant respondents, stratified by migrant status in the vignette.

**Results:**

Regarding the depression vignette depicting a non-migrant, there were only few differences between subgroups. However, when presented with a vignette describing someone from Turkey, respondents with migrant background who were foreign-born expressed greater stigmatizing attitudes, e.g. when it comes to stereotypes or desire for social distance. Furthermore, this subsample displayed higher levels of self-stigma of depression, especially regarding the ascription of own responsibility.

**Conclusions:**

The results underline the need to incorporate migration status/ethnicity in stigma research. Differences in attitudes as well as in (anticipated) self-stigma of depression identify foreign-born migrants in Germany as important target groups for tailored anti-stigma interventions, which need to consider diverse cultural backgrounds.

**Electronic supplementary material:**

The online version of this article (10.1186/s12888-017-1549-y) contains supplementary material, which is available to authorized users.

## Background

Mental health-related stigma is a multidimensional problem, which imposes a great burden on those affected. The stigma of mental illness can be defined as a process in which labeling, stereotyping, status loss and discrimination co-occur in a situation where power is exercised [1]. Public stigma of mental illness can be assessed by different components such as the ascription of attributes (stereotypes), emotional response upon meeting a person with mental disorder (e.g. anger, fear or pity) or the desire to socially distance oneself. Almost half of all public attitude research in Western countries focuses on depression [2]. In comparison to schizophrenia, public attitudes toward depression tend to be more positive [3]. Nevertheless, depression stigma has been found to be stable over the past two decades [4].

Referring to the ‘stigma complex’ as described by Pescosolido and Martin [5], interrelated structures and different social conditions have to be considered in order to gain a better understanding of the complicated nature of mental illness stigma. However, there are barely any studies which compare public beliefs and attitudes regarding psychiatric disorders across different cultures [2]. Furthermore, migration in relation to mental illness stigma has rarely been examined. This association can be considered from two perspectives. On the one hand, little is known about the prevalence and magnitude of mental illness stigma among migrants and in how far there might be differences when compared to non-migrants. On the other hand, it has rarely been explored whether migrants who are affected by mental illness are possibly exposed to double stigma.

Tabassum, Mackaskill, and Ahmad [6] attended to the mental illness stigma prevalent in a Pakistani community living in the United Kingdom. They found that none of the respondents would consider marriage to a person with mental illness, and only half agreed to socialize with such a person. A review of studies on stigma in Muslim communities came to the conclusion that there seems to be a pronounced mental illness stigma [7]. Due to concerns regarding the family’s social standing, disclosure of mental disorders is often considered ‘shameful’ [8], and there is an endorsement of stereotypes as well as support for segregation of psychiatric facilities from the community [9]. Regarding differences in mental illness stigma depending on the perceiver’s origin, Corrigan and Watson [10] explored the influence of ethnicity on the stigma of psychiatric disorders using vignettes. Compared to whites, the non-white group showed decreased likelihood to pity the health conditions and was more inclined to perceive someone with a mental health condition as dangerous. In the framework of an antistigma intervention, Rao, Feinglass, and Corrigan [11] also examined ethnic differences in stigmatizing attitudes. There were similar patterns prior and after the intervention, with Asians and African Americans perceiving a person with mental illness as more dangerous and expressing greater need for segregation than Caucasians.

In a prior study, von dem Knesebeck, Kofahl, and Makowski attended to the aspect of the potential burden of double stigma [12]. Following the concept of ‘layered stigma’ [13] or ‘multiple stigma’ [14] a person can belong to one or more possibly stigmatized groups, e.g. being mentally ill and of migrant background. This, in turn, might have additive effects on the individual [15]. The study [12] examined differences in attitudes toward migrants and non-migrants afflicted by depression. The respondents less often felt annoyed or pitiful when the person in the depression vignette was a migrant, and overall, migrants with depression did not seem to be confronted with double stigma. However, possible differences in public stigma depending on migrant status have not yet been examined in Germany.

Public stigma of mental illness has been found to be positively associated with self-stigma [16]. Furthermore, a non-disclosure out of fear of being stigmatized or due to self-stigma can lead to insufficient utilization of treatment, which in turn bears the risk of more serious courses of disease or chronification [17, 18].

Against this background, this study strives to explore attitudes regarding depression among migrants and non-migrants in Germany. In terms of migration status of the respondents, we use a definition by the Federal Office of Statistics, which includes all foreigners as well as people born in Germany with at least one foreign, naturalized or immigrated parent [19]. According to recent data, the share of individuals with migration background in Germany is 21% [20]. Regarding this current study, around 18% of all respondents had a migration background. One can assume that people with a migration background born in Germany display a relatively high level of acculturation when compared to those not born in Germany. Acknowledging this, we stratified our sample in three groups: non-migrants, migrants born in Germany and foreign-born migrants. First, differences between these three groups regarding three components of public stigma are analyzed: ascription of stereotypes, emotional responses, and desire for social distance [1, 21]. Furthermore, we test for variation in attitudes between migrants and non-migrants depending on the migration status in the vignette. Finally, the anticipated self-stigma of depression is compared between migrants and non-migrants.

## Methods

### Study design and sample

Analyses are based on a telephone survey (computer assisted telephone interview, CATI) which had been conducted in February and March 2016 in Germany. The sample consisted of the adult population (18 years and older) living in private households with access to a conventional telephone connection. Telephone numbers were drawn at random from all registered numbers. Additionally, computer-generated numbers were used to include ex-directory households. Within households, respondents were chosen via the Kish selection-grid [22], assuring that every person eligible for the interview has an equal chance to participate. Informed consent was considered to have been given when the interview was completed. In total, 2013 women and men voluntarily participated in the study, which reflects a response rate of 49%. The Ethics Commission of the Medical Association Hamburg approved the data collection procedure (No. PV3707). Comparison with official statistics showed that the distribution of gender and age is similar to that in the German public, while there is a slight overrepresentation of those with higher education (more than 12 years of education) in the present sample [12].

At the beginning of the interview, a vignette with typical signs and symptoms suggestive of depression was presented to the respondents (see Additional file 1). The survey was conducted by USUMA; a company specialized on market- and social research. During the CATI, the allocation of vignettes was carried out program-controlled to ensure an even and random distribution of vignette types. The vignette was developed with the input of experienced clinicians based on the Diagnostic and Statistical Manual of mental diseases (DSM IV) and the International Classification of Diseases (ICD 10) criteria and had also been used in previous research to elicit attitudes toward individuals with depression [23, 24]. In order to increase reliability and counteract possible interviewer effects, the vignette was audio-recorded by a trained speaker. This file was directly presented to the respondents from the computer via telephone line. Following the vignette, the respondents were asked to identify the disorder in the vignette. In case they did not know, they were informed that the person presented had a depression.

Of the 2013 respondents participating in the study, about 25% (*n* = 529) received a vignette describing a person with a Turkish name, stating that the person came from Turkey 4 years ago. Another 25% percent received the non-migrant vignette (*n* = 505). These subgroups were used for the analyses of differences in mental illness stigma. The remaining 50% of participants received either a depression vignette displaying a person with high or low socioeconomic status, which has been described elsewhere [12]. Regarding analyses of the self-stigma of depression, the total sample was used. The gender of the person in the vignette was also systematically varied, i.e. in 50% of the cases the person was female (please see Additional file 1 for vignettes). Rationale for choosing Turkey as country of origin in the vignette was that 2.9 million inhabitants in Germany are from Turkey. This constitutes the largest group of migrants in Germany, of which 61% solely hold Turkish citizenship, despite having settled here decades ago [25].

### Instruments

Three components of public stigma were assessed in the survey, which have also been defined by Link & Phelan [1] in the stigma process: ascription of stereotypes to a person with depression, emotional reactions upon meeting someone with depression, and desire for social distance. A list of six Likert items indicated stereotypes ascribed to a person with depression [26]. Using four response categories (ranging from 1 ‘completely disagree’ to 4 ‘completely agree’ plus ‘don’t know’ category), respondents were asked to indicate their (dis-)agreement with the following statements: ‘A person with depression is …’ ‘…in need of help’, ‘…unpredictable’, ‘…scary’, ‘…dangerous’, ‘…brilliant’, ‘…sensitive’.

Nine items represented different ways of emotionally responding to the person in the vignette. Each item was again coded from 1 ‘completely disagree’ to 4 ‘completely agree’. With these items, principal component analysis with varimax rotation was carried out and yielded the same three factors that had also been identified in previous research by Angermeyer and Matschinger [26]. The scale describing feelings of anger was comprised of the items ‘I react angrily’, ‘I feel annoyed by this person’, and ‘I react with incomprehension’. Cronbach’s α for the scale was 0.65. The items ‘This triggers fear’, ‘I feel uncomfortable’ and ‘I feel insecure’ loaded on the factor fear with a Cronbach’s α of 0.67. The items ‘I feel pity’, ‘I feel sympathy’, ‘I want to help’ constituted the third factor of pro-social emotions (Cronbach’s α = 0.51). Together, the three factors accounted for a cumulative variance of 58.6%. For each of the scales, sum scores were computed, ranging from 3 to 12.

A scale developed by Link et al. [27], which is a modified version of the Bogardus Desire for Social Distance scale [28], was used to measure the respondents’ desire for social distance from someone with depression. The scale is comprised of seven items that represent different social relationships: tenant, co-worker, neighbor, child carer, in-law, and person one would recommend for a job or person of the same social circle. Using a four-point Likert scale (ranging from 1 ‘totally agree’ to 4 ‘totally disagree’ plus ‘don’t know’ category), the respondents were asked to indicate to what extent they would, in the relationship presented, accept or not accept a person with a depression. With these seven items, a principal component analysis (with varimax rotation) was carried out, all items loaded on one factor explaining 42.05% of variance. Cronbach’s Alpha of the Desire for Social Distance scale was 0.77. A sum score (ranging from 7 to 28) was computed to assess the respondents’ overall desire for social distance.

Self-stigma of depression was assessed based on the Self-Stigma of Depression Scale (SSDS) [29, 30]. In the beginning, respondents were prompted as follows ‘Imagine, you were depressed. How would you feel?’ Then, they were presented with 16 different items, e.g. ‘I would feel inadequate around other people’ or ‘I would feel embarrassed about seeking professional help’. Answers could be given on a four-point Likert scale (ranging from 1 ‘completely disagree’ to 4 ‘completely agree’ plus ‘don’t know’ category). Principal component analysis with oblimin rotation [30] yielded three different factors of anticipated self-stigma of depression, including 10 out of the original 16 items: ‘Social Inadequacy’ (four items; Cronbach’s α = 0.77), ‘Own Responsibility’ (two items; Cronbach’s α = 0.48), and ‘Help-Seeking Inhibition’ (four items; Cronbach’s α = 0.71). Together, these factors accounted for a cumulative variance of 59.0%.

### Analyses

For categorical variables relative frequencies and for metric variables arithmetic means and standard deviations were computed. Independent analyses of variance (ANOVA) were applied to test for differences in stigmatizing attitudes toward either a migrant or non-migrant with depression between three groups of respondents: those with migrant background born in Germany, those with migrant background who were born in another country (foreign born) and respondents without migrant background. These analyses were adjusted for age, gender and education. To encounter multiple testing, Sidak correction was applied [31]. All statistical procedures were performed with the statistical package for the social sciences (SPSS 22) [32]. For the analyses of variance, partial η^2^ was computed. Exact *p*-values are reported for all analyses; the significance level is set at *p* < 0.05.

## Results

Sociodemographic characteristics of the respondents according to their migrant status are shown in Table 1. There were significant differences between the three groups regarding level of education and age. Respondents of migrant background were younger and had a higher educational level when compared to non-migrants. The groups did not differ with respect to gender. The subsequent analyses (results displayed in Tables 2, 3, 4 and 5) were adjusted for gender, age, and education level.

**Table 1:** Sociodemographic characteristics of the respondents split by migrant status

	Total sample (*N* = 1987)^*^	Migrant born in Germany (*n* = 139; 7.0%)	Migrant foreign born (*n* = 225; 11.3%)	Non-Migrant (*n* = 1622; 81.6%)	Differences subsamples p^a^
Sex (female) (%)	51.4	51.2	58.6	50.2	0.078
Level of education (%)					
*≤ 9 years*	36.1	28.3	35.3	36.8	*0.032*
*10 years*	32.1	34.1	29.0	32.4	
*≥ 12 years*	31.8	37.6	35.7	30.8	
Age (Mean (SD))	50.6 (18.3)	43.9 (18.9)	46.5 (19.1)	51.8 (17.9)	*<0.001*

^a^Kruskal-Wallis; *of the 2013 respondents, 26 had to be excluded from further analyses due to ambiguity of migrant status; statistically siginificant results (*p* < 0.05) are italicized

**Table 2:** Mean values (standard errors) of ascription of stereotypes towards someone with depression depending on respondents’ migration background and migration status in the depression vignette

Item	Non-migrant^a^ (*n* = 644–708)	Migrant born in Germany^a^ (*n* = 58–62)	Migrant foreign born^a^ (*n* = 98–101)	Results of analyses of variance (F, partial η^2^, *p*)
Ascription of stereotypes^b^ The person in the vignette is…				
…in need of help				
Non-migrant vignette	3.30 (0.04)	3.62 (0.13)	3.35 (0.10)	F_(2,429)_ = 2.772, η^2^ = 0.013, *p* = 0.064
Migrant vignette	3.32 (0.03)	3.55 (0.10)	3.45 (0.09)	F_(2,469)_ = 2.846, η^2^ = 0.012, *p* = 0.059
…unpredictable				
Non-migrant vignette	2.52 (0.05)	2.54 (0.16)	2.32 (0.13)	F_(2,419)_ = 0.983, η^2^ = 0.005, *p* = 0.375
Migrant vignette	2.49 (0.05)	2.39 (0.15)	2.46 (0.09)	F_(2,426)_ = 0.224, η^2^ = 0.001, *p* = 0.799
…scary				
Non-migrant vignette	1.96 (0.05)	2.07 (0.15)	2.21 (0.12)	F_(2,419)_ = 2.090, η^2^ = 0.010, *p* = 0.125
Migrant vignette	1.82 (0.04)	1.98 (0.12)	2.28 (0.11)^**c**^	F_(2,452)_ = 8.179, η^2^ = 0.035, *p* *< 0.001*
…dangerous				
Non-migrant vignette	1.97 (0.04)	2.26 (0.15)	1.77 (0.12)^d^	F_(2,419)_ = 3.539, η^2^ = 0.017, *p* = *0.030*
Migrant vignette	2.02 (0.04)	2.03 (0.12)	2.24 (0.12)	F_(2,446)_ = 1.643, η^2^ = 0.007, *p* = 0.195
…brilliant				
Non-migrant vignette	2.20 (0.05)	2.25 (0.16)	2.37 (0.13)	F_(2,393)_ = 0.796, η^2^ = 0.004, *p* = 0.452
Migrant vignette	2.09 (0.04)	2.42 (0.13)^**c**^	2.07 (0.12)	F_(2,420)_ = 3.212, η^2^ = 0.015, *p* = *0.041*
…sensitive				
Non-migrant vignette	3.33 (0.04)	3.60 (0.13)	3.40 (0.10)	F_(2,426)_ = 2.091, η^2^ = 0.010, *p* = 0.215
Migrant vignette	3.34 (0.03)	3.53 (0.10)	3.34 (0.09)	F_(2,465)_ = 1.694, η^2^ = 0.007, *p* = 0.185

^a^Mean values adjusted for gender, age and level of education; statistically significant results (*p* < 0.05) are italicized

^b^Ascription of stereotypes items ranging from 1 ‘totally disagree’ to 4 ‘totally agree’

^c^significantly different from non-migrant

^d^significantly different from migrant born in Germany

**Table 3:** Mean values (standard errors) of emotional reactions towards someone with depression depending on respondents’ migration background and migration status in the depression vignette

Item	Non-migrant^a^ (*n* = 644–708)	Migrant born in Germany^a^ (*n* = 58–62)	Migrant foreign born^a^ (*n* = 98–101)	Results of analyses of variance (F, partial η^2^, *p*)
Emotional reactions Upon meeting this person…				
…I react angrily.^b^				
Non-migrant vignette	1.45 (0.03)	1.59 (0.11)	1.33 (0.09)	F_(2,427)_ = 1.839, η^2^ = 0.009, *p* = 0.160
Migrant vignette	1.43 (0.03)	1.49 (0.10)	1.59 (0.09)	F_(2,461)_ = 1.433, η^2^ = 0.006, *p* = 0.240
…I feel annoyed.^b^				
Non-migrant vignette	1.87 (0.04)	2.00 (0.15)	1.73 (0.12)	F_(2,426)_ = 1.090, η^2^ = 0.005, *p* = 0.337
Migrant vignette	1.73 (0.04)	1.78 (0.12)	1.64 (0.11)	F_(2,449)_ = 0.412, η^2^ = 0.002, *p* = 0.662
…I react with incomprehension.^b^				
Non-migrant vignette	1.64 (0.04)	1.79 (0.14)	2.04 (0.11)^d^	F_(2,425)_ = 5.796, η^2^ = 0.027, *p* = *0.003*
Migrant vignette	1.63 (0.04)	1.51 (0.12)	2.00 (0.11)^d,e^	F_(2,462)_ = 5.923, η^2^ = 0.025, *p* = *0.003*
Scale Anger^c^				
Non-migrant vignette	4.96 (0.09)	5.37 (0.31)	5.05 (0.25)	F_(2,428)_ = 0.835, η^2^ = 0.004, *p* = 0.435
Migrant vignette	4.79 (0.08)	4.77 (0.26)	5.16 (0.24)	F_(2,460)_ = 1.093, η^2^ = 0.005, *p* = 0.336
…I feel pity.^b^				
Non-migrant vignette	3.09 (0.04)	3.08 (0.14)	3.36 (0.11)	F_(2,428)_ = 2.709, η^2^ = 0.012, *p* = 0.068
Migrant vignette	3.01 (0.04)	3.28 (0.13)	3.19 (0.12)	F_(2,466)_ = 1.871, η^2^ = 0.008, *p* = 0.155
…I feel sympathy.^b^				
Non-migrant vignette	2.68 (0.05)	2.82 (0.16)	2.64 (0.12)	F_(2,396)_ = 0.433, η^2^ = 0.002, *p* = 0.649
Migrant vignette	2.67 (0.04)	2.83 (0.13)	2.56 (0.11)	F_(2,428)_ = 1.223, η^2^ = 0.006, *p* = 0.295
…I feel the need to help.^b^				
Non-migrant vignette	3.20 (0.04)	3.14 (0.13)	3.47 (0.11)^d^	F_(2,426)_ = 3.099, η^2^ = 0.014, *p* = *0.046*
Migrant vignette	3.12 (0.03)	3.22 (0.11)	3.13 (0.09)	F_(2,467)_ = 0.345, η^2^ = 0.001, *p* = 0.708
Scale Prosocial^c^				
Non-migrant vignette	9.01 (0.09)	9.04 (0.31)	9.43 (0.24)	F_(2,430)_ = 1.350, η^2^ = 0.006, *p* = 0.260
Migrant vignette	8.83 (0.08)	9.43 (0.25)	8.83 (0.22)	F_(2,465)_ = 2.542, η^2^ = 0.011, *p* = 0.080
…I feel uncomfortable.^b^				
Non-migrant vignette	2.03 (0.05)	1.94 (0.16)	1.97 (0.13)	F_(2,420)_ = 0.221, η^2^ = 0.001, *p* = 0.802
Migrant vignette	2.00 (0.04)	1.70 (0.15)	2.50 (0.13)^d,e^	F_(2,451)_ = 9.339, η^2^ = 0.040, *p* < *0.001*
…he/she scares me.^b^				
Non-migrant vignette	1.69 (0.04)	1.51 (0.14)	1.74 (0.11)	F_(2,426)_ = 0.844, η^2^ = 0.004, *p* = 0.431
Migrant vignette	1.55 (0.03)	1.57 (0.11)	1.63 (0.09)	F_(2,465)_ = 0.327, η^2^ = 0.001, *p* = 0.721
…I feel insecure.^c^				
Non-migrant vignette	1.89 (0.05)	1.87 (0.17)	2.03 (0.13)	F_(2,429)_ = 0.490, η^2^ = 0.002, *p* = 0.613
Migrant vignette	1.85 (0.04)	1.74 (0.13)	1.94 (0.12)	F_(2,465)_ = 0.664, η^2^ = 0.003, *p* = 0.515
Scale Fear^b^				
Non-migrant vignette	5.61 (0.12)	5.37 (0.39)	5.67 (0.30)	F_(2,439)_ = 0.211, η^2^ = 0.001, *p* = 0.810
Migrant vignette	5.39 (0.10)	5.04 (0.31)	5.89 (0.27)	F_(2,469)_ = 2.349, η^2^ = 0.010, *p* = 0.097

^a^Mean values adjusted for gender, age and level of education; statistically significant results (*p* < 0.05) are italicized

^b^Emotional reaction items ranging from 1 ‘totally disagree’ to 4 ‘totally agree’

^c^Emotional reaction scales ranging from 3 to 12, comprised of 3 items (sum scores)

^d^significantly different from non-migrant

^e^significantly different from migrant born in Germany

**Table 4:** Mean values (standard errors) of desire for social distance from someone with depression depending on respondents’ migration background and migration status in the depression vignette

Item	Non-migrant^a^ (*n* = 644–708)	Migrant born in Germany^a^ (*n* = 58–62)	Migrant foreign born^a^ (*n* = 98–101)	Results of analyses of variance (F, partial η^2^, *p*)
Desire for Social Distance				
Tenant^b^				
Non-migrant vignette	2.40 (0.05)	2.56 (0.18)	2.31 (0.14)	F_(2,420)_ = 0.592, η^2^ = 0.003, *p* = 0.554
Migrant vignette	2.48 (0.05)	2.15 (0.16)	2.51 (0.14)	F_(2,452)_ = 2.145, η^2^ = 0.009, *p* = 0.118
Neighbor^b^				
Non-migrant vignette	1.69 (0.04)	1.65 (0.14)	1.72 (0.11)	F_(2,427)_ = 0.071, η^2^ = 0.000, *p* = 0.931
Migrant vignette	1.67 (0.04)	1.71 (0.12)	1.93 (0.10)	F_(2,462)_ = 2.943, η^2^ = 0.013, *p* = 0.054
Colleague^b^				
Non-migrant vignette	1.67 (0.04)	1.43 (0.13)	1.68 (0.10)	F_(2,429)_ = 1.650, η^2^ = 0.008, *p* = 0.193
Migrant vignette	1.67 (0.04)	1.81 (0.13)	1.67 (0.10)	F_(2,460)_ = 0.786, η^2^ = 0.003, *p* = 0.456
Childcare^b^				
Non-migrant vignette	3.05 (0.05)	2.78 (0.16)	3.08 (0.13)	F_(2,420)_ = 1.321, η^2^ = 0.006, *p* = 0.268
Migrant vignette	2.88 (0.05)	2.89 (0.14)	3.23 (0.13)^d^	F_(2,448)_ = 3.897, η^2^ = 0.017, *p* = *0.021*
In-law^b^				
Non-migrant vignette	2.25 (0.05)	2.27 (0.16)	2.65 (0.13)^d^	F_(2,403)_ = 4.284, η^2^ = 0.021, *p* = *0.014*
Migrant vignette	2.18 (0.05)	2.37 (0.14)	2.89 (0.13)^d,e^	F_(2,426)_ = 13.316, η^2^ = 0.059, *p* *< 0.001*
Introduce a friend^b^				
Non-migrant vignette	2.14 (0.05)	2.17 (0.16)	2.20 (0.12)	F_(2,421)_ = 0.105, η^2^ = 0.000, *p* = 0.900
Migrant vignette	2.21 (0.05)	2.21 (0.15)	2.40 (0.13)	F_(2,454)_ = 0.988, η^2^ = 0.004, *p* = 0.373
Job recommendation^b^				
Non-migrant vignette	2.59 (0.05)	2.54 (0.16)	2.56 (0.13)	F_(2,407)_ = 0.049, η^2^ = 0.000, *p* = 0.952
Migrant vignette	2.57 (0.05)	2.46 (0.14)	2.62 (0.13)	F_(2,454)_ = 0.361, η^2^ = 0.002, *p* = 0.697
Desire for Social Distance Scale^c^				
Non-migrant vignette	15.75 (0.21)	15.39 (0.72)	16.24 (0.57)	F_(2,214)_ = 0.487, η^2^ = 0.002, *p* = 0.615
Migrant vignette	15.65 (0.20)	15.52 (0.62)	17.23 (0.54)^d^	F_(2,462)_ = 3.979, η^2^ = 0.017, *p* = *0.019*

^a^Mean values adjusted for gender, age and level of education; statistically significant results (*p* < 0.05) are italicized

^b^Desire for Social Distance single items ranging from 1 ‘totally agree’ to 4 ‘totally disagree’

^c^Desire for social distance scale comprised of 7 items, total score ranging from 7 to 28 (sum score)

^d^significantly different from non-migrant

^e^significantly different from migrant born in Germany

**Table 5:** Self-Stigma of Depression Scale: Single items and scales according to respondents’ migration background [mean values (standard errors)]

Imagine you were depressed. How would you feel?	Migrant foreign born^a^ (*n* = 139)	Migrant born in Germany^a^ (*n* = 225)	Non-migrant^a^ (*n* = 1622)	Results of analyses of variance (F, partial η^2^, *p*)
Subscale Social Inadequacy	2.57 (0.02)	2.61 (0.06)	2.67 (0.05)	F_(2,1728)_ = 1.645, η^2^ = 0.002, *p* = 0.193
*Feel inferior to other people*	2.55 (0.03)	2.50 (0.09)	2.61 (0.07)	F_(2,1726)_ = 0.574, η^2^ = 0.001, *p* = 0.563
*Feel like a burden to other people*	2.82 (0.02)	2.94 (0.08)	2.82 (0.06)	F_(2,1737)_ = 1.057, η^2^ = 0.001, *p* = 0.348
*Feel inadequate around other people*	2.31 (0.02)^b^	2.46 (0.08)	2.47 (0.07)	F_(2,1683)_ = 3.539, η^2^ = 0.004, *p* = *0.029*
*Feel I couldn’t contribute much socially*	2.59 (0.02)^b^	2.58 (0.08)	2.79 (0.07)	F_(2,1728)_ = 4.236, η^2^ = 0.005, *p* = *0.015*
Subscale Own Responsibility	2.76 (0.02)^b^	2.89 (0.06)	3.02 (0.05)	F_(2,1667)_ = 11.295, η^2^ = 0.013, *p* *< * *0.001*
*Think I should be able to pull myself together*	2.77 (0.02)^b^	2.89 (0.08)	3.07 (0.07)	F_(2,1733)_ = 7.411, η^2^ = 0.008, *p* = *0.001*
*Think I should be able to cope with things*	2.75 (0.02)^b^	2.86 (0.08)	2.97 (0.06)	F_(2,1698)_ = 6.179, η^2^ = 0.007, *p* = *0.002*
Subscale Help-Seeking Inhibition	2.03 (0.02)	2.06 (0.06)	2.14 (0.05)	F_(2,1767)_ = 2.213, η^2^ = 0.002, *p* = 0.110
*Wouldn’t want people to know that I wasn’t coping*	2.24 (0.02)	2.24 (0.08)	2.37 (0.07)	F_(2,1765)_ = 2.146, η^2^ = 0.002, *p* = 0.117
*See myself as weak if I took antidepressants*	2.14 (0.13)	2.35 (0.09)	2.18 (0.07)	F_(2,1727)_ = 2.886, η^2^ = 0.003, *p* = 0.056
*Feel embarrassed about seeking professional help*	1.79 (0.02)	1.79 (0.08)	1.93 (0.07)	F_(2,1766)_ = 2.157, η^2^ = 0.002, *p* = 0.116
*Feel embarrassed if others knew I was seeking professional help*	1.97 (0.03)	1.88 (0.09)	2.06 (0.07)	F_(2,1757)_ = 1.340, η^2^ = 0.002, *p* = 0.262

^a^Mean values adjusted for gender, age and level of education;  statistically significant results (*p* < 0.05) are italicized; single items and scales ranging from 1 to 4 (‘completely disagree’-‘completely agree’)

^b^significantly different from non-migrant

Regarding the six items that capture stereotypes ascribed to someone with depression (Table 2), there was one significant difference between the subsamples when presented with a non-migrant vignette. Foreign-born migrants perceived such a person as less dangerous than migrants born in Germany. If the person presented in the depression vignette was from Turkey, foreign-born migrants significantly more often rated her or him as being scary when compared to non-migrants. The ascription of brilliance was more pronounced among migrants born in Germany.

Emotional reactions upon meeting an individual with depression are displayed in Table 3. Regardless of the depression vignette presented, respondents who were foreign-born stated they would react with incomprehension significantly more often than non-migrants or migrants born in Germany. When presented with a migrant vignette, foreign-born migrants displayed significantly greater feelings of discomfort than the other two subgroups. Regarding the depression vignette presenting a person from Germany, foreign-born migrants expressed more feelings to help this person.

Regarding desire for social distance (Table 4), respondents with migrant background born in Germany and those without migrant background did not differ significantly when presented with the two depression vignettes. However, when presented with a depression vignette depicting someone from Turkey, those of migrant background born abroad displayed significantly greater desire for social distance. This held true for the sum score as well as for the item childcare. Greatest desire for social distance was elicited by the hypothetical situation to accept a person with depression as an in-law. Compared to the other two subgroups, foreign-born migrants would not accept a German person with depression; the vignette depicting someone from Turkey induced even greater reluctance.

In terms of anticipated self-stigma, five statistically significant differences emerged (Table 5). In comparison to non-migrants, foreign-born migrants rather agreed to the feeling of inadequacy when around others as well as to the inability to contribute socially. Moreover, they tended to show greater self-blame in all aspects. There were no statistically significant differences between migrants born in Germany and non-migrants.

As displayed in Tables 2, 3, 4 and 5, the effect sizes of the statistically significant results were small to moderate.

## Discussion

The present study analyzed differences in stigmatizing attitudes towards a person with depression depending on the respondent’s migration background as well as migration status presented in the vignette.

When presented with a non-migrant vignette of a person suffering from depression, there were only few significant differences between respondents depending on their migration status with respect to the ascription of stereotypes and desire for social distance. In comparison with the other two subgroups, those of migrant background born abroad perceived a non-migrant with depression as less dangerous, and they were more reluctant to accept such a person as an in-law. Regarding emotional reactions, foreign-born migrants displayed significantly more incomprehension. When presented with a depression vignette depicting someone from Turkey, further statistically significant differences emerged. Regardless of the vignette, non-migrant respondents displayed relatively similar attitudes and largely this was true for migrants born in Germany as well. However, respondents of migrant background born abroad tended to hold more stigmatizing views. They agreed more often to the stereotype of a person with depression being ‘scary’ and displayed greater feelings of discomfort. Furthermore, pronounced differences emerged in the desire to distance oneself from someone with depression who is of migrant background. With respect to the anticipated self-stigma of depression, those of migrant background born abroad showed more feelings of inadequacy and especially greater self-blame when compared to non-migrants.

Previous international research has shown that the stigma of mental illness is highly prevalent among some groups of migrants [10, 11]. A study among a Muslim community found prejudices, which may hinder the disclosure of mental illness, as it is regarded very shameful [8]. Furthermore, there is a great reluctance to socially engage with individuals with mental illness, as it has been shown by Tabassum, Macaskill, and Ahmad [6]. This also holds true for the sample in the present study. To our knowledge, this is the first study in Germany examining differences in stigmatizing attitudes not only between migrants and non-migrants, but also depending on the migrant status in a vignette used as stimulus for the interview.

Many people of migrant background have been living in Germany for decades, e.g. based on recruitment contracts in the 1960s, concluded to encounter a deficit in workforce. Those migrants settled in Germany for good, and their descendants are living here in second or third generation. With reference to the present study, one could argue that many respondents of migrant background identify as members of the majority population, as they have been born or living here for the better part of their lives. This implies that their primary socialization has taken place in Germany. Although one can assume that their parents may have retained large parts of the heritage culture, values and norms passed on to their children could have already been adjusted to those of the receiving culture. During secondary socialization, friends, teachers, colleagues or media additionally shape attitudes, values and behaviors. Regarding attitudes toward persons with depression, this may explain why those of migrant background born in Germany do not substantially differ from non-migrants. Deviations from sociocultural or behavioral norms constitute the basis of labeling a condition as mental illness, which shows that mental illness is deeply rooted in culture and may vary between cultures [11]. Research has shown that the German public is informed quite well about depression [33], and that depression is less stigmatized than other psychiatric disorders (e.g. schizophrenia) [23]. However, people with migrant background born abroad may have been socialized with different beliefs of mental illness. A review by Fabrega [34] has shown that already in preindustrial Arab cultures, ‘insanity’ was a highly private matter handled by and rather kept within the family. Furthermore, studies have shown that in some cultures, explanatory models of mental illness include beliefs in supernatural causes or spirits [9, 35], which shape attitudes toward those affected by mental illness and reinforce their exclusion.

In the present study, most pronounced differences emerged with regard to desire for social distance. This corroborates findings of previous studies, which indicate a strong social stigma surrounding mental illness among migrant communities. Especially acceptance of a person with depression as an in-law was relatively low, and other studies have also found that revealing mental illness can jeopardize a marital relationship [9]. These social aspects of mental illness stigma are also expressed in levels of self-stigma among migrants in this study. The significant differences between subgroups underline the perceived shame related to depression, expressed in greater feelings of inadequacy around others. Furthermore, respondents of migrant background were more likely to see personal responsibility for the disorder, indicating self-blame.

As there are significant differences between the subsamples regarding sociodemographics (age and level of education), the analyses of depression attitudes have been adjusted for these variables. Stigmatizing attitudes have been found to display positive associations with age [36] as well as negative associations with educational attainment [37]. Interestingly, the migrant respondents are on average younger than the non-migrants. Moreover, in comparison to the non-migrant group, a greater share of migrant respondents attained the highest level of education. Nevertheless, stigmatizing attitudes persist.

When evaluating our findings, some limitations have to be mentioned and discussed. The response rate in this survey was about 50%. This is considered quite good for telephone surveys in Germany [38], however, we cannot rule out a selection bias due to non-response. Nevertheless, the comparison of sociodemographic data of our sample with official German statistics is satisfactory and supports the study’s validity [12]. A further limitation is related to the assessment of migrant background in our sample. We are not able to ascertain for how long respondents born abroad have been living in Germany, or from which country they originate. This aspect is not negligible in terms of acculturation and social identification. Furthermore, we presented a vignette displaying someone of Turkish background. Although this constitutes the largest group of migrants in Germany, countries of origin of the respondents (or their parents) certainly vary. The attitudes elicited by the vignette may also be influenced by other factors than solely by the diagnosis of depression. Regarding the vignette, it has to be mentioned that only the first sentence was varied to describe the different personal backgrounds of the persons displayed (see Additional file 1). Maybe this stimulus was not strong enough to convey the group affiliation, or the vignette was too short for respondents in order to develop a holistic picture of the person displayed. However, research has shown that vignettes should be kept rather short [39]. In terms of statistical analyses, it can be regarded critically that Likert scales were tested using parametric measures. However, non-parametric measures do not allow testing for variation between adjusted mean values, which we thought important regarding the differences obtained in sociodemographic variables between subsamples. Previous studies by Glass et al. [38] showed that the F-test is extremely robust to violations of its assumptions, and controls the Type I error rate well under conditions of skewness, kurtosis and non-normality. However, this does not apply for the assumption of homogeneity of variance, which was tested using Levene’s test. In those cases where the assumption was violated, non-parametrical Kruskal-Wallis-tests (results not displayed) were used to crosscheck the results. With one exception concerning the item ‘incomprehension’, we were able to verify the results obtained in ANOVA, which contributed to the decision to report these instead of the results of non-parametrical tests.

## Conclusions

To our knowledge, this is the first study in Germany that does not only consider differences in attitudes depending on migrant status of the respondent, but also of the person presented in the vignette. On an international level, only few studies examined possible ethnic differences in attitudes toward mental illness, and the present study contributes to the literature in this area. The results suggest that migrant background can have an impact on shaping stigmatizing views on mental illness and those afflicted. Moreover, the present study shows that foreign-born migrants display greater levels of self-stigma. Self-stigma has been found to be positively associated with hospitalization rates [40] and negatively associated with help-seeking [41]. This underlines the need for considering ethnicity in stigma research. It is of importance to gain a better understanding of stigmatizing processes, which take place depending on socialization and different cultural origins. This would allow for a development of tailored anti-stigma measures based on the participant’s background.

## Additional file

Additional file 1: Vignettes. (DOCX 11 kb)

## Abbreviations

ANOVA: Analysis of variance
CATI: Computer assisted telephone interview
DSM IV: Diagnostic and statistical manual of mental diseases (4th edition)
ICD 10: International classification of diseases (10th edition)
SPSS: Statistical package for the social sciences
SSDS: Self-stigma of depression scale
