# Supplementary material for: Depression stigma and migration – results of a survey from Germany
Source: BMC Psychiatry. 2017 Nov 29;17:381. doi: 10.1186/s12888-017-1549-y (PMC5707908; doi:10.1186/s12888-017-1549-y)
Supplement: Additional file 1: — Vignettes. (DOCX 11 kb) [file 12888_2017_1549_MOESM1_ESM.docx]

**Vignettes**

Migrant vignette:

46-year-old **Gülsen D.**^*^ came to Germany from Turkey four years ago. She has been constantly downhearted and unhappy for the last few months. She worries about the future. Mrs. D. feels useless, has the impression everything she does is wrong and has lost all interest in everyday activities. Besides, she complains about insomnia and feels nerveless and weak, already in the mornings. Mrs. D’s capability to work turns out to be declining.

Non-migrant vignette:

46-year-old **Dagmar D.**^*^ has been constantly downhearted and unhappy for the last few months. She worries about the future. Mrs. D. feels useless, has the impression everything she does is wrong and has lost all interest in everyday activities. Besides, she complains about insomnia and feels nerveless and weak, already in the mornings. Mrs. D’s capability to work turns out to be declining.

^*^Gender of the person in the depression vignettes was systematically varied.
